# Fusogenic Hybrid Extracellular Vesicles with PD-1 Membrane Proteins for the Cytosolic Delivery of Cargos

**DOI:** 10.3390/cancers14112635

**Published:** 2022-05-26

**Authors:** Raga Ishikawa, Shosuke Yoshida, Shin-ichi Sawada, Yoshihiro Sasaki, Kazunari Akiyoshi

**Affiliations:** Department of Polymer Chemistry, Graduate School of Engineering, Kyoto University, Katsura, Nishikyo-ku, Kyoto 615-8510, Japan; ishikawa.raga.3t@kyoto-u.ac.jp (R.I.); ssk-yoshida@bs.naist.jp (S.Y.); sawada@bio.polym.kyoto-u.ac.jp (S.-i.S.); sasaki@bio.polym.kyoto-u.ac.jp (Y.S.)

**Keywords:** extracellular vesicle, baculovirus expression system, liposome, membrane fusion, programmed cell death 1, cytosolic delivery

## Abstract

**Simple Summary:**

We developed a method using cell-derived lipid membrane capsules—called extracellular vesicles (EVs)—to deliver a model cargo into cytosol. These EVs were fused with liposomes (to form hybrid EVs) because cargo molecules can be more easily encapsulated within liposomes than EVs. EVs were engineered that expressed programmed cell death 1 (PD-1) and baculoviral envelope glycoprotein (gp64), which enabled the hybrid EVs to be internalized in cells and fuse with acidic organelles. The model cargo, Texas Red-labeled dextran, was shown to be released to the cytosol from the hybrid EVs by fusion with acidic organelles, such as late endosomes and lysosomes. Thus, these hybrid EVs are potential drug delivery carriers.

**Abstract:**

Extracellular vesicles (EVs) are cell-derived lipid membrane capsules that can deliver functional molecules, such as nucleic acids, to target cells. Currently, the application of EVs is limited because of the difficulty of loading cargo into EVs. We constructed hybrid EVs by the fusion of liposomes and insect cell-derived EVs expressing recombinant programmed cell death 1 (PD-1) protein and baculoviral fusogenic glycoprotein gp64, and evaluated delivery of the model cargo molecule, Texas Red-labeled dextran (TR-Dex), into the cytosol. When PD-1 hybrid EVs were added to HeLa cells, the intracellular uptake of the hybrid EVs was increased compared with hybrid EVs without PD-1. After cellular uptake, the PD-1 hybrid EVs were shown to be localized to late endosomes or lysosomes. The results of fluorescence resonance energy transfer (FRET) indicated that membrane fusion between the hybrid EVs and organelles had occurred in the acidic environment of the organelles. When TR-Dex-loaded liposomes were fused with the PD-1 EVs, confocal laser scanning microscopy indicated that TR-Dex was distributed throughout the cells, which suggested that endosomal escape of TR-Dex, through membrane fusion between the hybrid EVs and acidic organelles, had occurred. These engineered PD-1 hybrid EVs have potential as delivery carriers for biopharmaceuticals.

## 1. Introduction

Intracellular transport is an essential process for the delivery of drugs that function in the cytosol, such as mRNA vaccines [1]. In addition, mRNA, siRNA, and other nucleic acid drugs require DDS carriers that can efficiently deliver drugs to the cytosol because the of the low stability and membrane permeability of the naked drugs [2]. These drugs taken into the target cell by endocytosis must escape the endosomal barrier and enter the cytosol. Therefore, it is necessary for the carrier to have the ability to permeate or disrupt the endosomal membrane.

Extracellular vesicles (EVs) are cell-derived lipid membrane capsules that deliver functional molecules, such as proteins and nucleic acids, to target cells [3]. EVs have been studied for use as carriers in drug delivery systems (DDSs) because of their high cargo transportability [4]. Although EVs have shown certain effects, such as improved delivery to target sites, their application is limited because of the difficulty of loading the delivery cargo into EVs [5]. The most common method of loading cargo into EVs is via electroporation, which often induces denaturation and aggregation of the cargo [6]. Thus, an efficient method for loading cargo, such as drugs, into EVs is desirable. In addition, some drugs need to be delivered to the cytosol and to specific organelles, such as the nuclei and mitochondria, which requires the endosomal escape of the drugs loaded into EVs [7]. Therefore, it is important to impart endosomal escape functions to the EVs, for instance, by modifying the EV surface with a cell-penetrating peptide [8,9] or with a positive charge [10]. Previous studies have demonstrated that engineered EVs can effectively deliver siRNA to target cells and tissues in vitro and in vivo [11,12]. However, there are many unknown factors along the pathways required for siRNAs to reach the cytosol of target cells, and there are still considerable challenges in controlling the cytosolic delivery of EV cargos [13].

Membrane fusion, such as intracellular organelle fusion [14], cell–cell fusion [15], and virus–host cell fusion [16,17], is important in living systems. Many factors can cause membrane fusion, including proteins [18], peptides [19], and synthetic polymers such as polyethylene glycol [20,21]. Viruses effectively use envelope proteins as tools to deliver their genomes into host cells [22]. Enveloped viruses, such as the influenza virus and vesicular stomatitis virus, require membrane fusion between the viral membrane and the plasma or endosomal membranes to deliver the viral genome into the cytosol of host cells [16,17]. This viral fusion process is regulated by envelope proteins that induce membrane fusion by protein structural changes in response to decreased pH values and/or by binding to a receptor [16,17]. Therefore, viral envelope proteins are promising functional molecules for cytosolic drug delivery.

**Figure 1:**
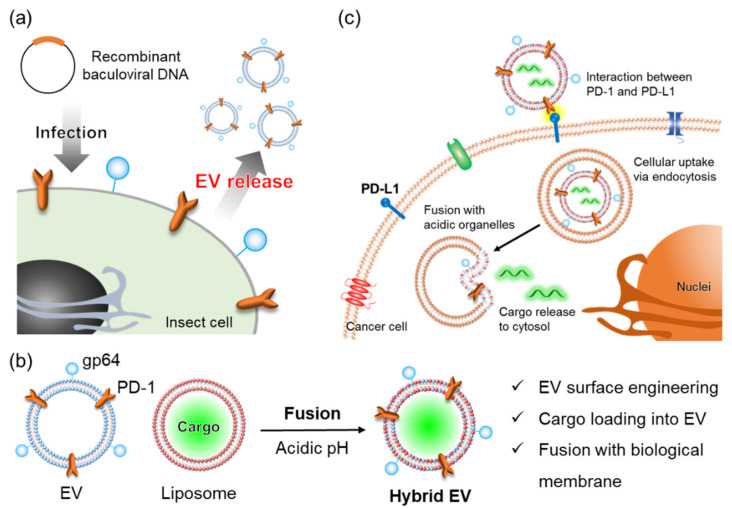
Schematic illustration of the construction and method of action of the engineered EVs. (**a**) Preparation of insect Sf9-derived EVs equipped with recombinant membrane proteins. (**b**) PD-1 hybrid EVs were prepared by membrane fusion between PD-1 EVs and liposomes under acidic conditions via the fusogenic protein gp64. (**c**) Delivery of PD-1 hybrid EV cargos to the cytosol by membrane fusion between the PD-1 hybrid EVs and acidic organelles.

The baculovirus expression system is a very useful protein production technique which uses the baculoviruses that infect insect cells [23]. We have recently developed engineered EVs derived from insect cells that contain recombinant programmed cell death 1 (PD-1) membrane proteins using a baculovirus expression system (Figure 1a) [24]. Recombinant PD-1 was effectively presented on insect cell-derived EV surfaces. Due to the specific binding of PD-1 to the ligand PD-L1, these PD-1 EVs were internalized into HeLa cells, which are PD-L1-expressing cancer cells [24]. Furthermore, the baculoviral envelope glycoprotein gp64, which mediates membrane fusion under acidic conditions, was also expressed on the PD-1 EVs [24]. When baculoviruses are taken up by host insect cells, the nucleocapsid is released into the cytosol by gp64-mediated membrane fusion induced by the decreased pH in the endosomes [25,26]. Viral gp64 is expressed on host insect cells infected with baculoviruses, and budded viruses acquire the gp64-expressing host cell membrane as an envelope [25]. Since gp64 can also mediate membrane fusion with artificial lipid membrane vesicles (liposomes) [27,28,29,30], here, we prepared hybrid EVs by fusing PD-1 EVs with cargo-loaded liposomes (Figure 1b). It is relatively easy to load a cargo inside liposomes as compared with EVs [31]; therefore, various cargo molecules can be introduced into hybrid EVs by fusing cargo-loaded liposomes with EVs. In addition, the functions of the liposomal membrane mean that the surface properties of hybrid EVs (e.g., charge, labeling, and colloidal stability) can be controlled [32,33]. It was expected that the interaction between PD-1 and PD-L1 would promote intracellular uptake of the PD-1 hybrid EVs into PD-L1-expressing cancer cells via endocytosis. This uptake would be followed by cargo release into the cytosol by gp64-mediated fusion between the PD-1 hybrid EVs and acidic organelles, such as late endosomes and lysosomes (Figure 1c). Therefore, these fusogenic hybrid EVs functionalized with PD-1 and gp64 membrane proteins were expected to be useful for intracellular drug delivery and to provide a new perspective for future EV engineering.

## 2. Materials and Methods

### 2.1. Materials

The Sf9 insect cell line derived from the fall armyworm *Spodoptera frugiperda*, commonly used in the baculovirus expression system, was purchased from Invitrogen (Waltham, CA, USA). HeLa cells were purchased from JCRB Bank (Japanese Collection of Research Biosources, Osaka, Japan); 1,2-Dioleoyl-sn-glycero-3-phosphocholine (DOPC), 1,2-dioleoyl-sn-glycero-3-phospho-L-serine (DOPS), 1,2-dioleoyl-sn-glycero-3-phosphoethanolamine-*N*-(cyanine 5) (Cy5-DOPE), 1,2-dioleoyl-sn-glycero-3-phosphoethanolamine-*N*-(7-nitro-2-1,3-benzoxadiazol-4-yl) (NBD-DOPE), and 1,2-dioleoyl-sn-glycero-3-phosphoethanolamine-*N*-(lissamine rhodamine B sulfonyl) (Rho-DOPE) were purchased from Avanti Polar Lipids (Alabaster, AL, USA).

### 2.2. Construction of Recombinant Baculoviruses

The recombinant baculoviruses encoding the PD-1 mutant were constructed using the Bac-to-Bac baculovirus expression system previously described [24]. Briefly, a pFastBac1 plasmid with the membrane protein sequence of interest was transformed into DH10Bac *Escherichia coli*, which contained a bacmid and helper plasmid encoding the transposase gene. The target gene inserted between Tn7 transposon sequences was transposed into the bacmid. Colonies containing the recombinant bacmid were identified by blue/white selection and the bacmid was isolated using a PureLink HiPure Plasmid Miniprep Kit (Invitrogen). Sf9 cells were transfected with the recombinant bacmid using Cellfectin II Reagent (Invitrogen) and incubated at 27 °C for 5 days. The supernatant containing P1 viruses was collected, and the viral concentration was amplified three times. The viral titers were determined by a BacPAK Baculovirus Rapid Titer Kit (Takara Bio USA, Inc., San Jose, CA, USA).

### 2.3. Isolation of Extracellular Vesicles

The method for isolation of the Sf9-derived EVs was the same as previously reported [24]. Briefly, 4.0 × 10^5^ cells/mL Sf9 cells were maintained in Sf-900 III serum-free medium (Invitrogen) overnight at 27 °C. Budded virus suspension was added at a multiplicity of infection of 0.5 and incubated at 27 °C for 96 h. This infection step induced the expression of the target recombinant proteins and viral gp64 proteins on host cells. The culture medium was centrifuged at 500× *g* for 5 min and 2000× *g* for 10 min, at 4 °C, followed by 0.22 µm filtration. The supernatant was ultra-centrifuged at 100,000× *g* for 70 min at 4 °C and the resultant pellet was resuspended in phosphate-buffered saline (PBS). The suspension was ultra-centrifuged at 40,000× *g* for 30 min at 4 °C along a stepwise sucrose density gradient (10, 15, 20, 25 and 30% (weight/volume) in PBS buffer). The upper fraction, which contained the EVs, and the lower fraction, which contained the budded viruses, were collected separately. The protein concentration in the EVs was estimated using a Pierce BCA protein assay kit (Thermo Fisher Scientific, Waltham, MA, USA).

### 2.4. Liposome Preparation

DOPC, DOPS, NBD-DOPE, Rho-DOPE, and Cy5-DOPE were mixed in chloroform in glass microtubes at various molar ratios. The solvent was evaporated under flowing argon gas, resulting in the formation of a lipid film. The film was placed in a desiccator in vacuo overnight to completely remove the chloroform. The film was hydrated by adding 250 µL of buffer (20 mM CH_3_COOH/CH_3_COONa (pH 4.5) or 10 mM Tris-HCl (pH 7.5)) and incubated overnight at 27 °C. The suspension was extruded through a 100 nm pore polycarbonate membrane using a mini-extruder (Avanti Polar Lipids). The lipid concentration was measured using the Phospholipid C-Test (Wako, Osaka, Japan). Briefly, hydrogen peroxide was produced from phospholipid samples by phospholipase D and choline oxidase. The generated hydrogen peroxide promoted the condensation reaction of N-Ethyl-N-(2-hydroxy-3-sulfopropyl)-3,5-dimethoxyaniline (DAOS) and 4-aminoantipyrine in the presence of peroxidase, producing a blue dye. By measuring the absorbance of this dye, the phospholipid concentration in the sample was determined.

### 2.5. Nanoparticle Tracking Analysis

The size distributions of the EVs (0.1 µg/mL protein) and hybrid EVs (0.1 µM lipid) were measured using a NanoSight LM10 (NanoSight, Amesbury, UK). The samples were measured with a 532 nm wavelength laser at 25 °C and analyzed by NanoSight NTA 2.3 software.

### 2.6. Transmission Electron Microscopy

The suspension of EVs was placed on a copper grid coated with a formvar membrane for 5 min. After removing the suspension, 1% phosphotungstic acid solution was placed on the grid for 5 min and then removed. Samples were observed using an HT7700 transmission electron microscope (Hitachi, Tokyo, Japan) at an accelerating voltage of 100 kV.

### 2.7. Western Blotting

EV and hybrid EV samples solubilized with sodium dodecyl sulfate (SDS) buffer were separated using 12.5% polyacrylamide gel and transferred to polyvinylidene difluoride (PVDF) membranes. After blocking with PVDF Blocking Reagent for Can Get Signal (TOYOBO Co., Ltd., Osaka, Japan), the membranes were reacted with primary antibodies to PD-1 (ab89828, Abcam, Cambridge, UK) or gp64 (sc-65499, Santa Cruz Biotechnology, CA, USA). After washing with Tris-buffered saline with 0.1% Tween 20 (TBST), the membranes were reacted with horseradish peroxidase-conjugated goat anti-mouse IgG (Santa Cruz Biotechnology). After washing with TBST again, the membranes were reacted with ECL Western blotting detection reagents (GE Healthcare, Chicago, IL, USA) and the band signals were visualized using a LAS-4000 (GE Healthcare).

### 2.8. Imaging Flow Cytometry

PD-1 EVs were stained with 5- or 6-(*N*-succinimidyloxycarbonyl)fluorescein 3′,6′-diacetate (CFSE). CFSE was added to a PD-1 EV suspension to a final concentration of 62.8 µM, followed by incubation for 30 min at 37 °C. To remove free CFSE, the labeled PD-1 EVs were washed using 100,000 NMWL Amicon Ultra Centrifugal Filters (Merck Millipore, Burlington, MA, USA). Liposomes were prepared at a 100:100:1 molar ratio of DOPC, DOPS, and Cy5-DOPE. CFSE PD-1 EVs (5 µg/mL) and Cy5 liposomes (1 µM) were mixed under acidic or neutral conditions and incubated for 30 min at 27 °C. The fusion reaction was stopped by addition of pH 7.5 buffer in the same volume as the reaction solution. The PD-1 hybrid EVs were extruded through a 100 nm pore membrane. Multispectral images of PD-1 hybrid EVs were acquired by an ImageStream^x^ MkII (Merck Millipore). The laser powers were set to maximum (488 nm: 200 mW; 642 nm: 150 mW; and 785 nm (side scatter): 70 mW). Fluorescence signals were collected in channel 2 (CFSE) or channel 5 (Cy5). Channels 1 and 6 were set to brightfield and side scatter, respectively. Fluorescence intensities of 10,000 particles were acquired at 40× magnification and analyzed using IDEAS 6.2 software.

### 2.9. Cellular Uptake of Hybrid Extracellular Vesicles

Liposomes were prepared containing a 100:100:1 molar ratio of DOPC, DOPS, and Rho-DOPE. The hydrated rhodamine liposomes were extruded through a 100 nm pore polycarbonate membrane using a mini-extruder. The rhodamine liposomes (100 µM lipid) and PD-1 EVs (30 µg/mL protein) were mixed and incubated for 30 min at 27 °C under acidic conditions (pH 4.5). The fusion reaction was stopped by the addition of pH 7.5 buffer in the same volume as the reaction solution. The PD-1 hybrid EVs were extruded again through a 100 nm pore membrane. The lipid concentration of PD-1 hybrid EVs was measured using the Phospholipid C-Test (Wako). HeLa cells were cultured in Dulbecco’s Modified Eagle Medium (Thermo Fisher Scientific) containing 10% fetal bovine serum and 1% penicillin–streptomycin at 37 °C in a 5% CO_2_ incubator. To assess the co-localization of PD-1 hybrid EVs and HeLa cell organelles, early endosomes, late endosomes, and lysosomes were green fluorescent protein (GFP)-labeled using CellLight™ reagent BacMam 2.0 (Thermo Fisher Scientific), which is a fusion construct of each organelle marker and GFP. Briefly, by adding 5 µL of the reagent to 1 × 10^4^ HeLa cells and culturing in a CO_2_ incubator overnight, each organelle was stained with GFP. PD-1 hybrid EVs diluted in Opti-MEM reduced serum medium (Thermo Fisher Scientific) were incubated with 1 × 10^4^ HeLa cells for 1 or 4 h. After washing with PBS three times, the cells were observed with a confocal laser scanning microscope, LSM780 (Carl Zeiss, Oberkochen, Germany).

### 2.10. Evaluation of the Fusion of Hybrid Extracellular Vesicles with Acidic Organelles

Fusion between hybrid EVs and acidic organelles was evaluated by fluorescence resonance energy transfer (FRET). Liposomes were prepared at a 100:100:4:1 molar ratio of DOPC, DOPS, NBD-DOPE, and Rho-DOPE. NBD (excitation at 460 nm, emission at 535 nm) and rhodamine (excitation at 560 nm, emission at 583 nm) were fluorescence donor and receiver molecules for FRET, respectively. The hydrated liposomes were extruded through a 100 nm pore polycarbonate membrane using a mini-extruder. The FRET liposomes (100 µM lipid) and PD-1 EVs (30 µg/mL protein) were mixed and incubated for 30 min at 27 °C under acidic conditions (pH 4.5). The fusion reaction was stopped by the addition of pH 7.5 buffer in the same volume as the reaction solution. The FRET PD-1 hybrid EVs were extruded again through a 100 nm pore membrane. PD-1 hybrid EVs diluted in Opti-MEM reduced serum medium (Thermo Fisher Scientific) were incubated with 1 × 10^4^ HeLa cells for 4 h in the presence or absence of 100 nM bafilomycin A1, which is often used as a proton pump inhibitor. After washing with PBS three times, the cells were observed with a confocal laser scanning microscope, LSM780 (Carl Zeiss). The fluorescence spectra were measured using the lambda mode, with excitation at 488 nm and emission at 504–653 nm (9 nm intervals). The fluorescence intensity of each wavelength was normalized by dividing by the maximum fluorescence intensity within each region.

### 2.11. Cytosolic Delivery of Hybrid Extracellular Vesicle Cargo

A lipid thin film composed of DOPC and DOPS at a 1:1 molar ratio was hydrated with 1 mg/mL Texas Red-labeled dextran (TR-Dex, 3000 MW) (Thermo Fisher Scientific) solution dissolved in 10 mM Tris-HCl (pH 7.5) and the solution was incubated overnight at 27 °C. The suspension was extruded through a 100 nm pore polycarbonate membrane using a mini-extruder. To remove free TR-Dex, the suspension was washed using a PD SpinTrap G-25 (GE Healthcare). The TR-Dex-encapsulated liposomes (100 µM lipid) and PD-1 EVs (30 µg/mL protein) were mixed and incubated for 30 min at 27 °C under acidic conditions (pH 4.5). The fusion reaction was stopped by the addition of pH 7.5 buffer in the same volume as the reaction solution. The suspension was extruded again through a 100 nm pore membrane. Late endosomes and lysosomes of HeLa cells were GFP-labeled using CellLight™ reagent BacMam 2.0 (Thermo Fisher Scientific). The TR-Dex-encapsulated PD-1 hybrid EVs diluted in Opti-MEM reduced serum medium (Thermo Fisher Scientific) were incubated with 1 × 10^4^ HeLa cells for 4 h in the presence or absence of 100 nM bafilomycin A1. After washing with PBS three times, the cells were observed by a confocal laser scanning microscope, LSM780 (Carl Zeiss). The co-localization coefficient was calculated from the ratio of all Texas Red pixels to Texas Red pixels co-localized with GFP.

### 2.12. Statistical Analysis

The experimental data were statistically evaluated using the Mann–Whitney non-parametric test or the two-tailed Welch’s *t*-test. An adjusted *p* < 0.05 was considered statistically significant. All statistical analyses were performed using GraphPad Prism 9 (GraphPad Software, Inc., San Diego, CA, USA).

## 3. Results

### 3.1. Preparation and Characterization of Hybrid Extracellular Vesicles

**Figure 2:**
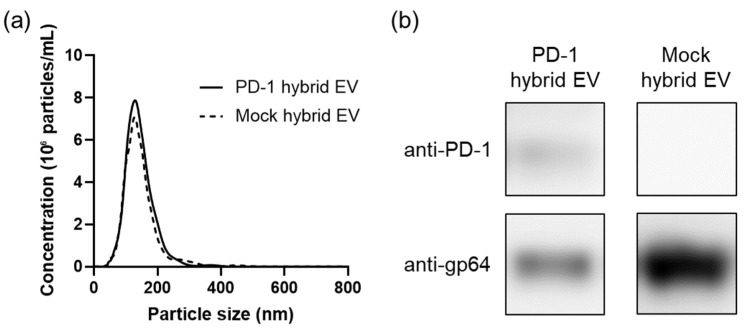
Characterization of hybrid EVs formed by fusion of EVs and liposomes under acidic conditions. (**a**) Size distributions of PD-1 hybrid EVs and mock hybrid EVs determined by NTA. (**b**) Western blot analysis of PD-1 hybrid EVs and mock hybrid EVs using antibodies against PD-1 and gp64.

PD-1 EVs were isolated from Sf9 cells that had been infected with recombinant baculoviruses encoding the PD-1 mutant gene. Mock EVs for use as controls were collected from Sf9 cells that had been baculovirus-infected by mock transfection. These Sf9-derived EVs were characterized by nanoparticle tracking analysis (NTA), Western blotting, and transmission electron microscopy (TEM) observations (Figure S1). Numerous EV-like particles with 100–200 nm particle diameters were identified (Figure S1a,c). Western blot analysis confirmed the expression of PD-1 in PD-1 EVs and of gp64 in PD-1 EVs and Mock EVs (Figure S1b). PD-1 hybrid EVs and mock hybrid EVs were prepared by mixing liposomes and PD-1 EVs or mock EVs under acidic conditions at 27 °C for 30 min. For later functional evaluation as DDS carriers, the hybrid EVs were extruded through a 100 nm filter. The size distributions of PD-1 hybrid EVs and mock hybrid EVs were determined by NTA (Figure 2a). The particle sizes of PD-1 hybrid EVs and mock hybrid EVs were 144 ± 44 and 144 ± 52 nm (mean ± SD), respectively. Western blot analysis indicated that PD-1 and gp64 were included in the PD-1 hybrid EVs (Figure 2b). In contrast, only gp64 was included in the mock hybrid EVs (Figure 2b). These results indicated that the membrane protein components of the EVs were retained in the hybrid EVs fused with liposomes.

**Figure 3:**
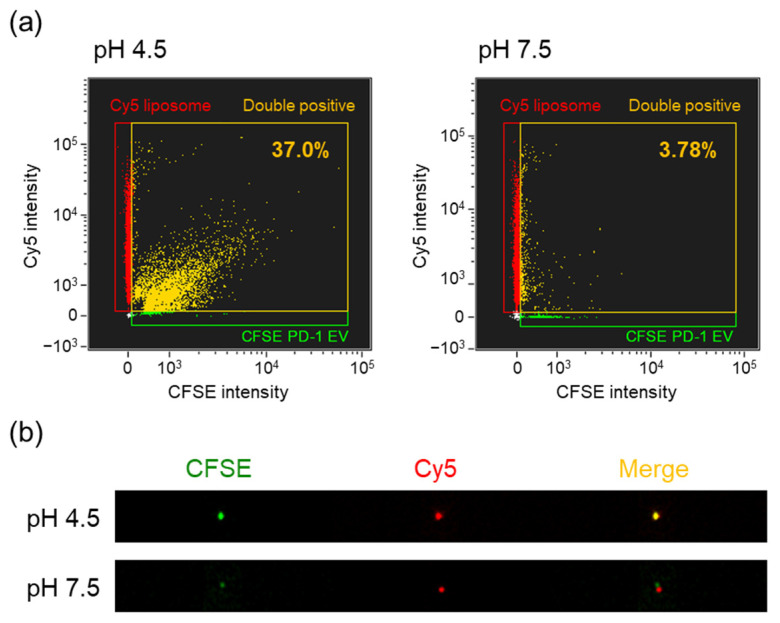
Single-particle fluorescence analysis of PD-1 hybrid EVs after extrusion. (**a**) Dot plots of PD-1 hybrid EVs prepared by fusion of CFSE-labeled PD-1 EVs (5 µg/mL) and Cy5-labeled liposomes (1 µM) at pH 4.5 or 7.5, as determined by IFC. Green, CFSE-single positive; red, Cy5-single positive; yellow, CFSE and Cy5 double-positive. (**b**) Representative fluorescence images of double-positive particles determined by IFC.

PD-1 EVs and liposomes were respectively labeled with CFSE and Cy5-DOPE and analyzed by imaging flow cytometry (IFC). Particles with CFSE and Cy5 fluorescence were indeed detected by IFC (Figure S2). CFSE-labeled PD-1 EVs were mixed with Cy5-labeled liposomes at pH 4.5 or 7.5 and the mixtures were extruded through a 100 nm filter. Then, the hybrid EVs were analyzed by IFC using the gating process shown in Figure S3. First, the target particle population was distinguished from the speed beads for flow control using a plot of side scatter intensity versus brightfield image area (Figure S3a). Next, the particles with an aspect ratio of zero for both CFSE and Cy5 fluorescent spots were determined to be fluorescent noise (Figure S3b). Finally, the population of particles remaining after removal of the fluorescent noise were subjected to analysis. Under acidic conditions, there was a particle population in the CFSE and Cy5 double-positive region (Figure 3a). Furthermore, the particle population was co-localized with CFSE and Cy5 fluorescence (Figure 3b). In contrast, under neutral conditions, there were hardly any particles populating the double-positive region (Figure 3a), and the particle population was not co-localized with CFSE and Cy5 (Figure 3b). These results indicated that the hybrid state was maintained even when the particle size of the hybrid EVs was reduced by the extrusion after fusion of EVs and liposomes under acidic conditions.

### 3.2. Intracellular Uptake and Organelle Localization of Hybrid Extracellular Vesicles

**Figure 4:**
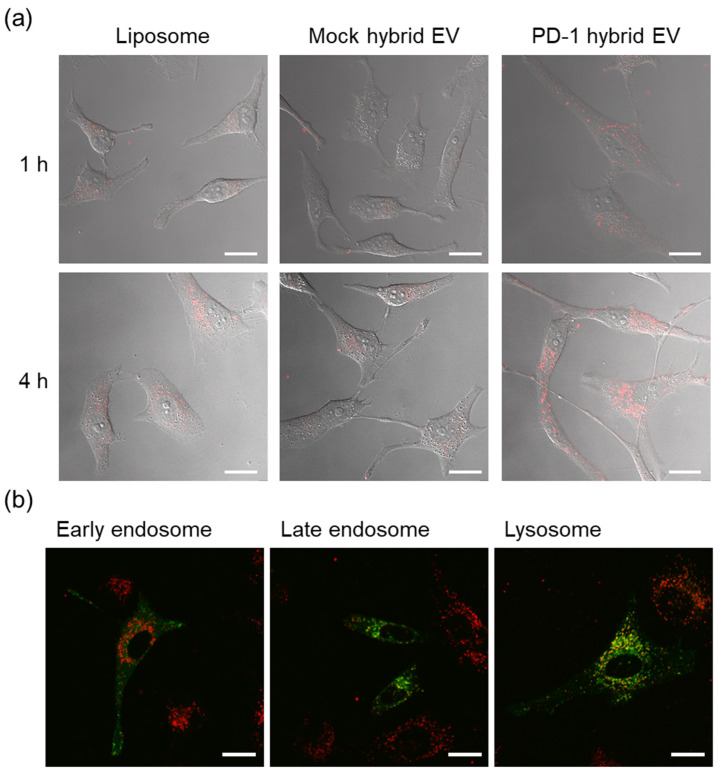
Intracellular uptake and organelle localization of PD-1 hybrid EVs labeled with rhodamine. Scale bars, 20 µm. (**a**) HeLa cells were incubated with rhodamine-labeled PD-1 hybrid EVs, mock hybrid EVs, or liposomes (12.5 µM lipid) for 1 or 4 h and observed by CLSM. (**b**) HeLa cells that had been pre-stained with GFP using CellLight™ reagents to detect early endosomes, late endosomes, and lysosomes were incubated with 12.5 µM PD-1 hybrid EVs for 4 h and observed with a CLSM.

Rhodamine-labeled PD-1 hybrid EVs were prepared by fusion with PD-1 EVs and DOPC/DOPS/Rho-DOPE liposomes under acidic conditions. Mock hybrid EVs were similarly prepared by fusion with mock EVs and rhodamine liposomes under acidic conditions. HeLa cells were incubated with PD-1 hybrid EVs, mock hybrid EVs, or rhodamine liposomes for 1 or 4 h, and then cellular uptake was observed using a confocal laser scanning microscope (CLSM). PD-1 hybrid EVs were more efficiently internalized in HeLa cells as compared with the control groups (liposomes and mock hybrid EVs), and the uptake increased with increasing incubation time (Figure 4a). We have previously reported that PD-1 EVs interact with PD-L1 protein and PD-L1-expressing HeLa cells [24]. The results of the present study indicated that the function of PD-1 EVs in interaction with HeLa cells was maintained after fusion with liposomes. In addition, early endosomes, late endosomes, and lysosomes of HeLa cells were stained with GFP before the addition of PD-1 hybrid EVs to evaluate the intracellular organelle localization of the incorporated PD-1 hybrid EVs. Four hours after addition of the PD-1 hybrid EVs to the HeLa cells, PD-1 hybrid EVs were transferred to late endosomes or lysosomes (Figure 4b and Figure S4). In contrast, 1 h after addition, hardly any localization of PD-1 hybrid EVs to late endosomes and lysosomes was observed (Figure S4). The pH in early endosomes is approximately pH 6.5, while the pH in late endosomes and lysosomes is more acidic (pH 4–5) [34]. Thus, PD-1 hybrid EVs transported to acidic organelles may fuse with the organelle membranes through the activation of gp64 in response to the low pH in the acidic organelles.

### 3.3. Membrane Fusion of Hybrid Extracellular Vesicles with Cell Organelles

**Figure 5:**
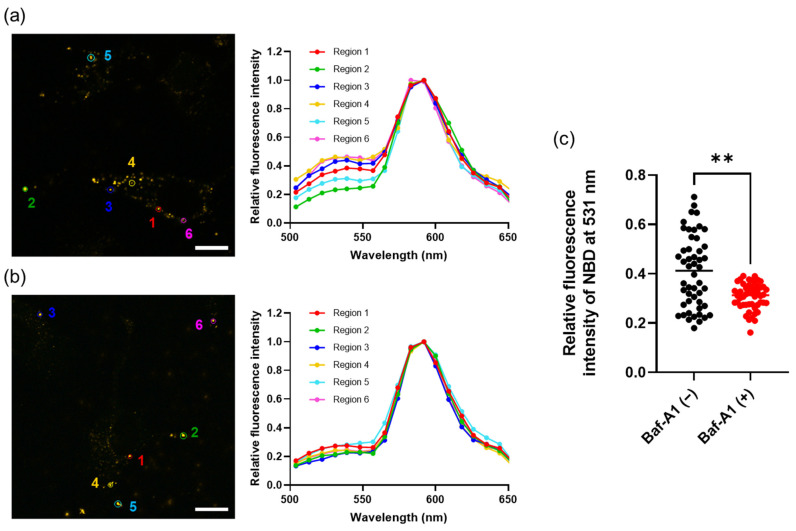
Interaction between the PD-1 hybrid EVs and acidic organelles. HeLa cells incubated with FRET PD-1 hybrid EVs in the absence (**a**) or presence (**b**) of Baf-A1 were observed using a CLSM in lambda mode. Scale bars, 20 µm. Representative fluorescence spectra were acquired from regions 1 to 6 of the CLSM images. (**c**) Relative intensity of NBD at 531 nm in the absence (black) or presence (red) of Baf-A1 was determined from 50 regions of CLSM images in three independent experiments. Each dot represents an individual value; the lines indicate the median values (** *p* < 0.01, Mann–Whitney non-parametric test).

The pH-dependent conformational switch of gp64 that triggers membrane fusion is reversible—a property that is not often found in other viral fusogenic proteins, except the vesicular stomatitis virus glycoprotein (VSV-G) [35]. Thus, the gp64 on PD-1 hybrid EVs prepared under acidic conditions may retain the fusion function. To evaluate the fusion of acidic organelles and PD-1 hybrid EVs, we prepared FRET PD-1 hybrid EVs fused with FRET liposomes. Membrane fusion between the FRET PD-1 hybrid EVs and acidic organelles should have caused a recovery of NBD fluorescence by FRET elimination. CLSM observations using the lambda mode revealed that the FRET PD-1 hybrid EVs were internalized in HeLa cells. Fluorescence spectra, with excitation set at 488 nm in regions 1–6 in the images, were obtained by CLSM observation in lambda mode. In some regions, the fluorescence intensity of NBD at 531 nm relative to the fluorescence intensity of rhodamine at 592 nm was increased (Figure 5a).

In addition, to assess whether the reduction of FRET was dependent on the acidic environment of the organelles, the proton pump inhibitor bafilomycin A1 (Baf-A1) was used. HeLa cells incubated with FRET PD-1 hybrid EVs in the presence of Baf-A1 were observed using a CLSM in lambda mode. In the presence of Baf-A1, the increase in the fluorescence intensity of NBD at 531 nm relative to rhodamine at 592 nm was suppressed compared with in the absence of Baf-A1 (Figure 5b,c). These results suggested that the FRET efficiency was reduced as a result of the membrane fusion of PD-1 hybrid EVs with acidic organelles, such as late endosomes and lysosomes, in HeLa cells.

### 3.4. Cargo Delivery to the Cytosol by Fusion between Hybrid Extracellular Vesicles and Acidic Organelles

**Figure 6:**
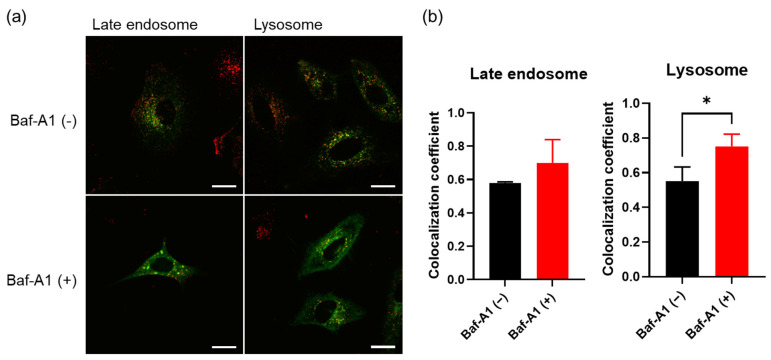
Evaluation of the cytosolic delivery of PD-1 hybrid EV cargo. (**a**) HeLa cells with pre-stained late endosomes and lysosomes were incubated with TR-Dex-encapsulated PD-1 hybrid EVs in the absence or presence of Baf-A1 and observed with a CLSM. Red indicates TR-Dex fluorescence; green indicates GFP fluorescence of the organelles. (**b**) The co-localization coefficient was calculated from the ratio of all rhodamine pixels to rhodamine pixels co-localized with GFP. Results are expressed as mean ± SD (* *p* < 0.05, two-tailed Welch’s *t*-test, *n* = 3).

Finally, the ability of PD-1 hybrid EVs to function as an intracellular delivery system was investigated. Texas Red-labeled dextran (TR-Dex) was encapsulated into liposomes by hydration of a DOPC/DOPS thin film (1:1 molar ratio) with TR-Dex solution. The TR-Dex-loaded liposomes fused with the PD-1 EVs under acidic conditions (pH 4.5). Late endosomes and lysosomes of HeLa cells were pre-stained with GFP, and TR-Dex-encapsulated PD-1 hybrid EVs were added to the HeLa cells in the presence or absence of Baf-A1. From the CLSM observations, in the absence of Baf-A1, TR-Dex was not co-localized with late endosomes or lysosomes and was distributed throughout the cells (Figure 6a). This result suggested that endosomal escape of TR-Dex through membrane fusion between hybrid EVs and acidic organelles had occurred. In contrast, in the presence of Baf-A1, TR-Dex was co-localized with the organelles (Figure 6a). The co-localization coefficient of TR-Dex for GFP-labeled organelles, calculated from the CLSM images, was increased in the presence of Baf-A1 (Figure 6b). Therefore, it can be speculated that the release of TR-Dex into the cytosol was due to the acidic nature of organelles such as late endosomes and lysosomes.

## 4. Discussion

As endosomes mature from early endosomes to late endosomes, the pH value decreases and eventually the endosomes fuse with lysosomes, resulting in the degradation of the materials within the endosomes [34]. The “proton sponge effect” is often exploited as an endosome escape method using the acidic environment of organelles [36]. This mechanism can explain the relatively efficient nucleic acid transfection by cationic polyplexes [37]. In brief, the buffering effect of the cationic carriers on endosomal pH causes an osmotic imbalance and the influx of water into the endosomes leads to membrane disruption [38]. The disruption of endosomal membranes by membrane-permeable peptides or cationic polymers has been proposed as another endosomal escape method [39,40,41]. In this case, the molecules interact directly with the endosomal membrane, causing membrane destabilization and subsequent membrane collapse [38]. Although some studies have reported successful gene delivery using these non-viral vector carriers, the delivery efficiency remains extremely low compared with that of viral vector carriers [38].

Envelope membrane proteins are important in the binding of viruses to host cells and in the fusion of plasma and endosomal membranes [42,43]. The conformational structures of envelope proteins are altered by binding to receptors or by a pH decrease, exposing hydrophobic peptide domains in the envelope proteins [44]. The interaction of this fusogenic region (hydrophobic domain) with plasma and endosomal membranes initiates membrane fusion with the envelope membrane and delivery of the viral genome into the cytosol is promoted [16,17]. The dynamic conformational changes of envelope proteins, such as hemagglutinin in the influenza virus [45] and VSV-G in the vesicular stomatitis virus [46], are tightly controlled by changes in pH. Therefore, the gene delivery efficiency of viral vectors with envelope proteins tends to be considerably higher than that of non-viral vectors such as polymer-based carriers [47].

In the present study, we developed hybrid EVs that fuse EVs and liposomes and evaluated the cytosolic delivery of the hybrid EV cargo by membrane fusion using the baculoviral envelope protein gp64. Although pH-dependent conformational changes of viral envelope proteins are usually irreversible, the conformational change of gp64 is reversible [35]. Thus, use of gp64 can result in multi-step membrane fusion. Therefore, we considered that EVs equipped with gp64 could be promising materials for the construction of cytosolic delivery systems using membrane fusion. Single-particle analysis by IFC confirmed the preparation of PD-1 hybrid EVs complexed with PD-1 EVs and liposomes. When these EVs were added to HeLa cells, the intracellular uptake of the PD-1 hybrid EVs was increased compared with mock hybrid EVs without PD-1. After cellular uptake, the PD-1 hybrid EVs localized to late endosomes or lysosomes, which are acidic organelles. These organelles then fused with the hybrid EVs in response to the acidic environment and the hybrid EV cargo was transferred to the cytosol. Since the surface composition of the hybrid EVs can be controlled by changing the mixing ratios of the EVs and liposomes under acidic conditions [30], the amount of PD-1 and gp64, the concentration of the cargo molecules, and the surface charge in the hybrid EVs can be controlled. By optimizing these factors, it is expected that increased control of the cytosolic delivery function of hybrid EVs will be possible in the future.

There were some limitations to this study. First, the toxicity of Sf9-derived EVs and hybrid EVs to HeLa cells was not evaluated. Baculovirus has been used as a gene vector capable of introducing an exogenous gene into not only insect cells but also a wide range of mammalian cells without replication [48,49]. In addition, the safety and immunogenicity of vaccine products obtained by the baculovirus expression system have also been investigated [50,51,52]. Similarly, PD-1 EVs used in this study also need to be evaluated in the future for their effects on the intrinsic functions of HeLa cells, such as intracellular uptake and intracellular transport. Second, only HeLa cells were used to evaluate the function of PD-1 hybrid EVs in cellular uptake and cytosolic delivery. We previously reported that PD-1 EVs derived from Sf9 cells bind to the PD-L1 ligand proteins and are incorporated into HeLa cells, which are PD-L1-expressing cancer cells [24]. Similarly, in this study, HeLa cells were first used to evaluate the function of PD-1 hybrid EVs. PD-L1 is highly expressed not only in HeLa cells but also in various types of cancer cells [53,54,55]. In addition, PD-L1 is also expressed not only in cancer cells but also in normal cells, such as immune cells, epithelial cells, and vascular endothelial cells [53,56]. Therefore, to demonstrate the tumor-selective delivery function of PD-1 hybrid EVs, various types of cancer cells and normal cells should be used in the future. Finally, only Texas Red-labeled dextran (TR-Dex) was used as a hybrid EV cargo. To prove the effective cytosolic delivery function of PD-1 hybrid EVs, it will be necessary to investigate whether molecules such as siRNA and mRNA can be delivered while retaining their functions.

## 5. Conclusions

We have developed membrane protein-engineered hybrid EVs that include PD-1 and gp64. The hybrid EVs targeted HeLa cells, a type of PD-L1-expressing cancer cell, and displayed fusogenicity with the acidic organelles. The hybrid EV cargo was released into the cytosol as a result of membrane fusion between the hybrid EVs and acidic organelles. The proof of concept of cytosolic delivery by EV protein engineering and membrane fusion in this study could expand the applicability of EVs as DDS carriers.

## Data Availability

The data that support the findings of this study are provided in the figures of this article.

## Supplementary Materials

The following supporting information can be downloaded at: https://www.mdpi.com/article/10.3390/cancers14112635/s1, Figure S1: (a) Size distributions of PD-1 EVs and mock EVs as determined by NTA. (b) Western blot analysis of PD-1 EVs and mock EVs using antibodies against PD-1 and gp64. (c) Morphologies of PD-1 EVs and mock EVs observed by TEM. Scale bars, 200 nm; Figure S2: Single-particle fluorescence analysis of PD-1 EVs and liposomes before membrane fusion. Dot plots of 5 µg/mL CFSE-labeled PD-1 EVs (a) and 1 µM Cy5-labeled liposomes (b) at pH 7.5 as determined by IFC; Figure S3: Gating process for detection of fluorescence nanoparticles by IFC. Plots were obtained in acidic conditions; the gating process was similar to that for neutral conditions. (a) Removal of speed beads using channels 1 (brightfield) and 6 (side scatter). (b) Removal of fluorescent noise for channels 2 and 5. Finally, 10,000 particles were acquired and analyzed in Population 2; Figure S4: HeLa cells that had been pre-stained with GFP using CellLight™ reagents to detect early endosomes, late endosomes, and lysosomes were incubated with 12.5 µM PD-1 hybrid EVs for 1 or 4 h and observed with a CLSM. Scale bars, 20 µm, Figure S5: Full Western Blots for Figure 2b and Figure S1b.
