# Supplementary material for: Fusogenic Hybrid Extracellular Vesicles with PD-1 Membrane Proteins for the Cytosolic Delivery of Cargos
Source: Cancers (Basel). 2022 May 26;14(11):2635. doi: 10.3390/cancers14112635 (PMC9179877; doi:10.3390/cancers14112635)
Supplement: Supplementary file 1 [file cancers-14-02635-s001.zip › cancers-1681486-supplementary.pdf]

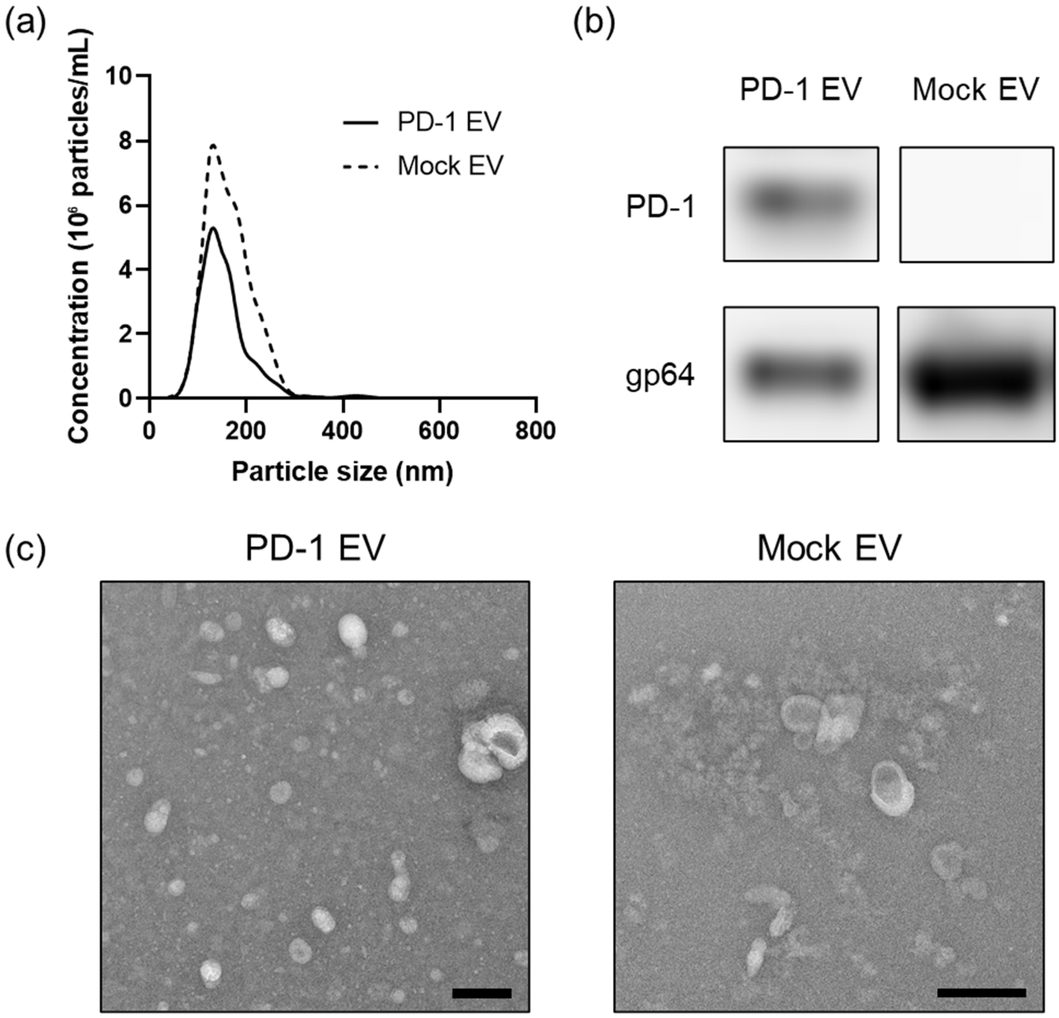

Figure S1. (**a**) Size distributions of PD-1 EVs and mock EVs as determined by NTA. (**b**) Western blot analysis of PD-1 EVs and mock EVs using antibodies against PD-1 and gp64. (**c**) Morphologies of PD-1 EVs and mock EVs observed by TEM. Scale bars, 200 nm.

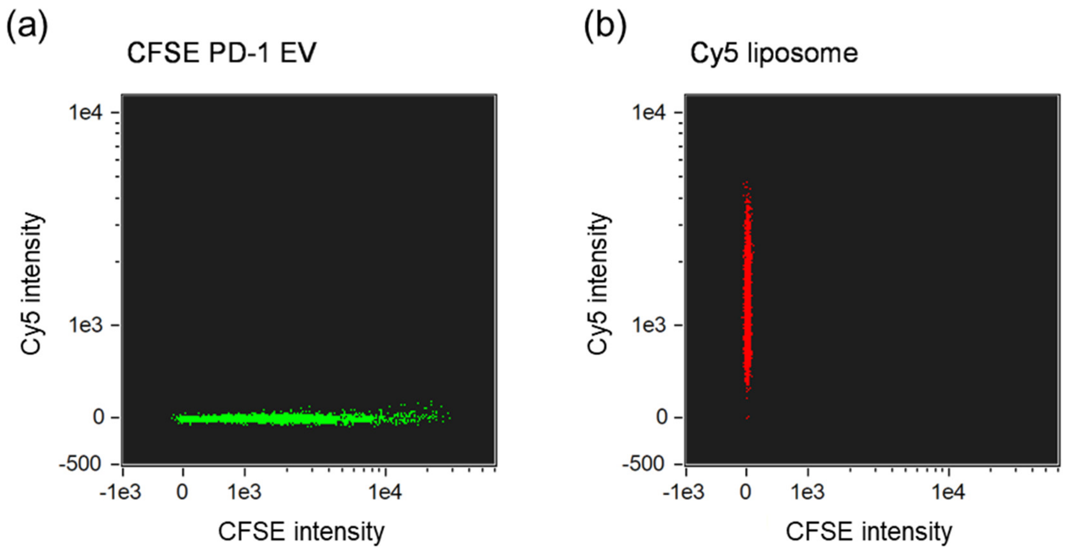

Figure S2. Single-particle fluorescence analysis of PD-1 EVs and liposomes before membrane fusion. Dot plots of 5 μg/mL CFSE-labeled PD-1 EVs (**a**) and 1 μM Cy5-labeled liposomes (**b**) at pH 7.5 as determined by IFC.

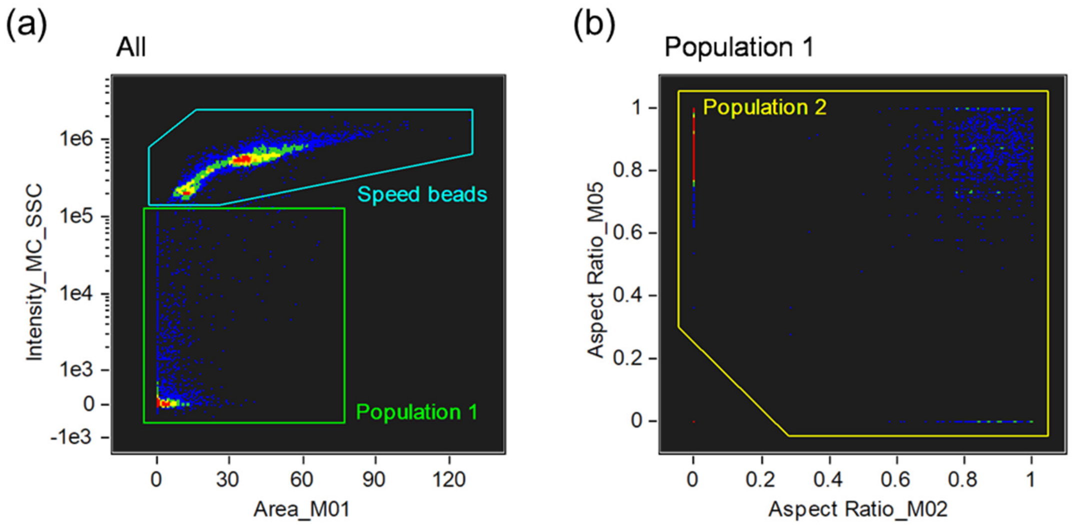

Figure S3. Gating process for detection of fluorescence nanoparticles by IFC. Plots were obtained in acidic conditions; the gating process was similar to that for neutral conditions. (**a**) Removal of speed beads using channels 1 (brightfield) and 6 (side scatter). (**b**) Removal of fluorescent noise for channels 2 and 5. Finally, 10,000 particles were acquired and analyzed in Population 2.

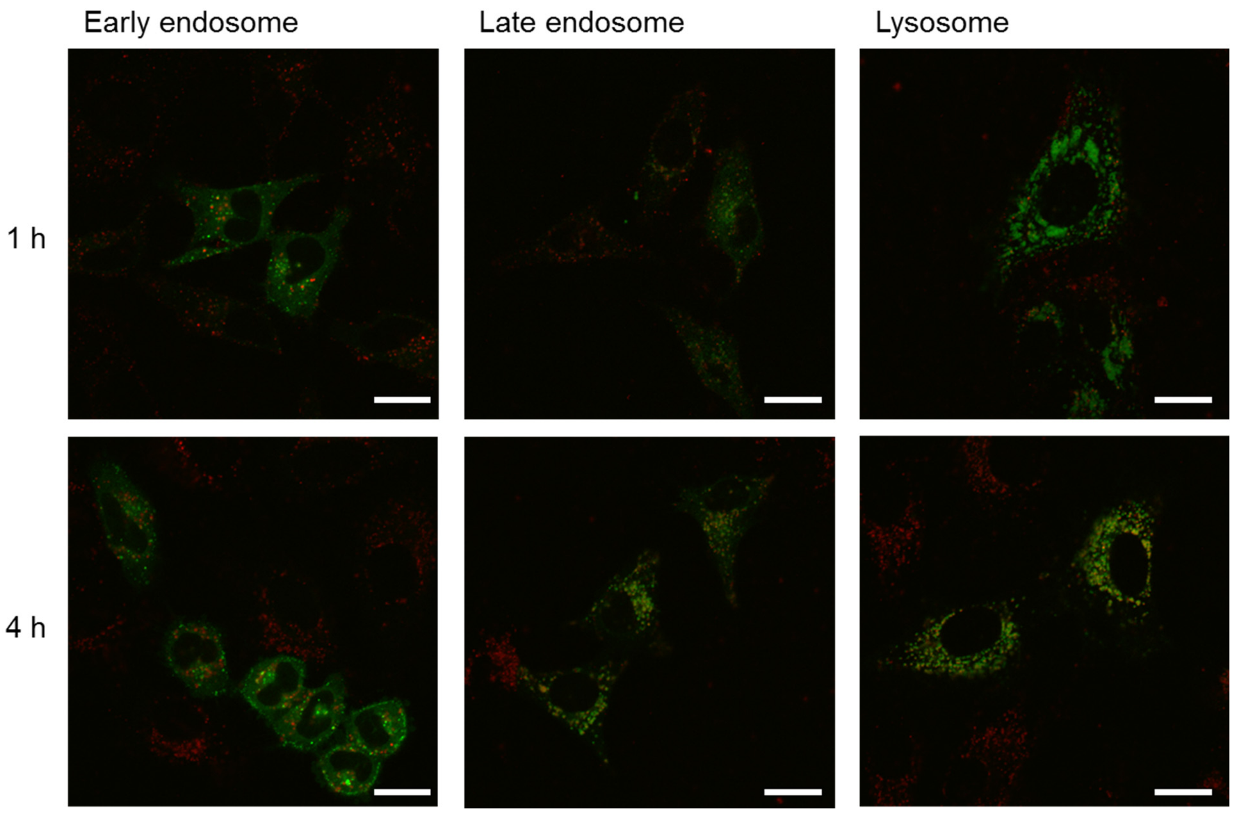

Figure S4. HeLa cells that had been pre-stained with GFP using CellLight™ reagents to detect early endosomes, late endosomes, and lysosomes were incubated with 12.5 μM PD-1 hybrid EVs for 1 or 4 h and observed with a CLSM. Scale bars, 20 μm.

①: PD-1 EV (Figure S1b)
②: PD-1 hybrid EV (Figure 2b)
③: Mock EV (Figure S1b)
④: Mock hybrid EV (Figure 2b)

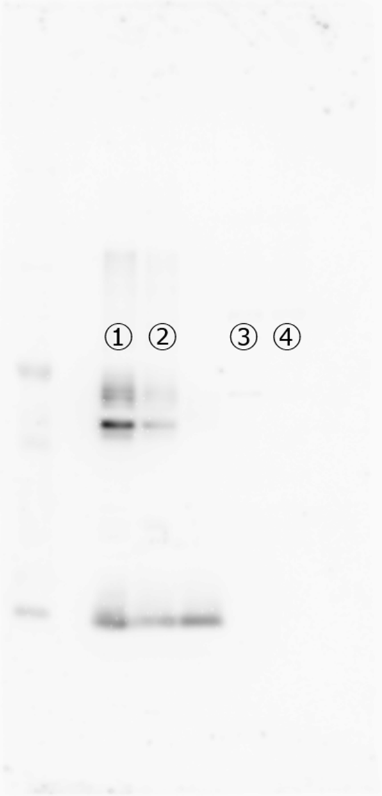

①: PD-1 EV (Figure S1b)
②: PD-1 hybrid EV (Figure 2b)
③: Mock EV (Figure S1b)
④: Mock hybrid EV (Figure 2b)

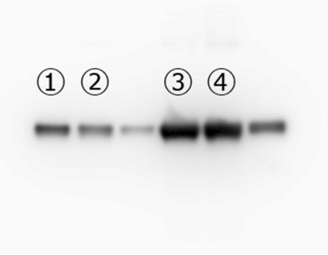

Figure S5. Full Western Blots for Figure 2b and S1b.
